# Addressing the Impact of Interpersonal Violence in Women Who Struggle with Substance Use Through Developmental-Relational Strategies in a Community Program

**DOI:** 10.3390/ijerph16214197

**Published:** 2019-10-30

**Authors:** Mary Motz, Naomi C. Z. Andrews, Bianca C. Bondi, Margaret Leslie, Debra J. Pepler

**Affiliations:** 1 Early Intervention Department, Mothercraft, 860 Richmond Street West, Toronto, ON M6J 1C9, Canada; mmotz@mothercraft.org (M.M.); mleslie@mothercraft.org (M.L.); 2 Department of Child and Youth Studies, Brock University, 1812 Sir Isaac Brock Way, St. Catharines, ON L2S 3A1, Canada; 3 Department of Psychology, York University, 4700 Keele Street, Toronto, ON M3J 1P3, Canada; bbondi@yorku.ca (B.C.B.); pepler@yorku.ca (D.J.P.)

**Keywords:** interpersonal violence, domestic violence, substance use, intervention, women, developmental–relational, gender-specific approach

## Abstract

From a developmental–relational framework, substance use in women can be understood as relating to early experiences of violence in relationships and across development. This article uses a developmental-relational approach to outline specific strategies that can be used by service providers and to guide interventions for women with substance use issues. By reviewing research and clinical work with women attending a community-based prevention and early intervention program, we describe how specific components of programming can target the developmental and intergenerational pathway between experiences of violence in relationships and substance use. We include the voices of women who attended the program to support the strategies discussed. Specifically, these strategies address the impact of interpersonal violence on substance use by promoting the process of repair and reintegration for women whose neurological development, sense of self, and capacity to form relationships have been significantly impacted by experiences of violence in relationships.

## 1. Introduction

Many women who struggle with substance use have had a history of violent and traumatic experiences in relationships, often beginning in childhood [1]. These early experiences of interpersonal violence can negatively impact both neuropsychological development (e.g., executive functioning and emotion regulation) and the development of relational capacity (e.g., attachment and internal working models) [2]. Some refer to interpersonal violence (IPV) as domestic violence, family violence, or intimate partner violence; we use the term IPV to highlight the intergenerational nature of violence in relationships and that violence in relationships is often not exclusive to violence between partners. A developmental-relational approach is one in which the bidirectional associations between development and relationships are emphasized as important processes in understanding behaviour and functioning. A developmental perspective emphasizes the transactional links between the individual and the environment, and the effect of each on the other over time [3]. From a relational perspective, we consider that people, institutions, and systems grow through relationships with others [4,5]. This perspective helps us understand the link between women’s experiences of violence in relationships across development and their later substance use issues. That is, through a developmental–relational approach, individuals’ behaviour (e.g., substance use) can be understood within a context that includes their history and development, with a particular focus on how behaviour is shaped through relationships. To be effective in supporting women to make changes and achieve their addiction recovery goals, interventions need to address the impact of disruptions to women’s development of self-regulation, executive functions, and relationship capacity as a result of their lifelong experiences with IPV [2]. Interventions need to be direct, purposeful, gender-specific, and trauma-informed, as well as grounded in attachment, developmental, and relational theory. With these theoretical and treatment foundations, it is possible to promote repair and reintegration for women whose neurological development, sense of self, and capacity to form healthy relationships have been significantly impacted by IPV.

Over the past 25 years, our research and clinical work with women attending a community-based prevention and early intervention program in Canada called Breaking the Cycle (BTC) have illuminated several specific strategies necessary to address the impact of IPV. Since 1995, BTC has provided comprehensive, integrated supports for mothers who are struggling with substance use issues, and their young children aged 0 to 6 years [6,7]. Programming at BTC is directed towards women, their children, and the mother–child relationship. BTC was developed through extensive community consultations that included street-based, community-based, and institutionally-based service providers who worked with women with substance use issues and substance-exposed children [8]. Interviews with members of the potential service population were also conducted. These consultations formed the basis of BTC’s design and philosophy. BTC operates under formal partnership with nine agencies that include services relating to addiction treatment, health, and child development and protection. The development of BTC has been an evolving and emergent process, and research and program evaluation have been built into BTC since its inception. Through evaluation and listening to the voices of the women and children served by BTC, the program has expanded its range of services as well as its methods of service delivery.

Research has identified components of intervention programming and the therapeutic relationship that women with substance use issues find supportive [9]. There is research pointing to how therapeutic relationships promote emotion regulation and executive functioning [10]. With research as an integral component of BTC, we have confirmed the efficacy of gender-specific interventions based on a developmental-relational approach for women with a history of IPV and substance use. We have demonstrated successful engagement of, and enduring service relationships with, this marginalized population of women [11,12] to achieve outcomes related to substance use recovery, mental health functioning, and relationship capacity [13]. The following section describes some key intervention strategies used at BTC to (1) promote self-regulation and executive functions and (2) promote safety and capacity in relationships. Focus groups and interviews are regularly conducted with women at BTC for program evaluation and research purposes. The quotes that follow are drawn from previous BTC program evaluations [6,8] and other interviews completed for research and program evaluation, as indicated. These quotes are provided to highlight the voices and perspectives of the women who attend BTC. All women provided consent for their words to be used in publication.

## 2. Promoting Self-Regulation and Executive Functions

IPV and early experiences of trauma and violence in relationships can have a profound impact on the development of self-regulation and executive functioning strategies [14,15]. Deficits in executive functions also pose a heightened risk for future substance use issues [16]. As such, from a developmental-relational perspective, promoting self-regulation and executive functions is an essential part of programming at BTC, particularly given that women who use substances as a result of lifelong experiences with IPV struggle to regulate their behaviour and emotions, which ultimately impacts their psychosocial functioning. To do this, programming at BTC focuses on: (1) supporting time management, (2) monitoring the organization and ambiance of the program space, (3) encouraging regulated interactions, and (4) paying attention to readiness and internal processes.

### 2.1. Supporting Time Management

*I like that she’ll meet me, just because it motivates you…For myself, being an addict, sometimes you need somebody to come to you, and help you…sometimes you’ll make appointments and cancel. And I’m fresh into recovery, and can’t wait to meet her there, it’s like she’s going to be there, she’s going to be there, which is really helpful because it’s motivating.*

[6] (p. 53)

When a woman makes the decision (whether assisted or unassisted) to call and access services for her substance use, it can be frustrating, disempowering, and demotivating to encounter an answering service or to be told that no one is available to meet with her for a number of weeks or months. During open hours at BTC, the telephone is always answered by a staff member and as soon as basic referral information is gathered, an initial appointment is scheduled within two weeks (two business days for pregnant women). Beginning with that first appointment, approaches and strategies are introduced to support women to be successful in accessing BTC services. For example, women with impaired executive functioning may struggle with organization and time management. Staff use appointment cards and/or provide women with calendars as tools to remind them of their next appointment. In scheduling appointments, BTC staff support women to consider a date and time that will work for them in the context of other priorities in their lives, which may include appointments with mandated services (e.g., probation or parole appointments, child welfare appointments), medical and mental health services, access visits with children, as well as child care/school drop-off and pick-up for children. In the early engagement phase of service, women are assured that their appointment time is reserved for them on a weekly basis and, even if they forget or are unable to attend, that appointment slot will be held for them in the following week. 

Throughout the entire time that a woman accesses service at BTC, there is predictability, not only with her own appointments, but also in the overall structure in which programming is delivered. Women are provided with reminder telephone calls about upcoming individual and group meetings as a means of supporting their time management, as well as helping them to recognize that staff are thinking about them and anticipating their presence at the centre. There is consistency and predictability in program scheduling. Groups are offered at the same day and time each week, year after year, and are wrapped around food services (daily breakfast and lunch); group interventions complement times when individual appointment sessions are offered and co-occur with child-minding hours. This service structure supports women to anticipate programming at BTC, to schedule their own time accordingly, and ultimately to increase the probability of them attending and achieving service plan goals.

### 2.2. Monitoring the Organization and Ambiance of the Program Space

*The whole package…(The Parent-Infant Therapist) still comes to our home on a bi-weekly basis, we have (the paediatrician/toxicologist) for any medical problems, we have (the public health nurse) for any questions on how to breastfeed…You don’t have to go to different locations all over the place, it’s all under one roof, so a lot of the information you can access here. And knowing that (the child development counsellors) are always there. Because you don’t have anyone else.*

[6] (p. 84)

The organization of the physical space at BTC has been carefully and deliberately planned. The integrated, single-access program is located in a downtown Toronto neighborhood which is accessible by public transportation. It is close to other services that BTC women access regularly, including withdrawal management and treatment services, community hospitals where there are physicians with expertise in addictions medicine, community housing and shelters, supplemental food programming, services for Indigenous peoples, and community healthcare.

BTC itself offers a wide range of programming so women can access many of the services that they need in one location. These services are offered at times that complement each other so that women do not have to participate in only one of two services that they need. Programming includes individual counselling and group interventions (e.g., addictions, mental health, interpersonal relationship, and trauma), intensive case management, daily breakfast and lunch, food and clothing donations, tokens for public transportation, prenatal street outreach, probation and parole appointments, parenting and early intervention programs for children ages 0 to 6 years, including weekly home visitation and child-minding.

The centre itself has a calm and home-like ambiance, which is often a contrast to the institutional settings where the women have accessed services previously. The main area of the centre is a spacious kitchen and living room where women are able to relax and attend to their own and their children’s needs. The sizable playroom enables women to participate in mother–child group programming and play with their children. It has natural lighting and a large internal window so women can observe their children. The centre has been designed to be comfortable and inviting, without being overstimulating for women (and children) who require support with self-regulation and executive functioning. Staff engage with the women and their children through friendly conversations in the main area of the centre. At the same time, BTC staff are conscientious about confidentiality and hold private counselling discussions in appropriate private spaces. BTC staff gently redirect conversation, when required, which models and promotes women’s self-regulation and boundary setting. BTC staff guide the women in a humble, relational, and positive way, without ever being punitive or shaming.

A significant component of supporting women with histories of IPV to develop a sense of trust in relationships is to provide safe and welcoming environments for them and for their children. At BTC, particular focus is given to ensuring that the environment feels safe to everyone who is in the centre. Programs are structured and drop-in services are not available; BTC staff know everyone who uses the space. Women who access programming at BTC know that staff are aware of and are familiar with everyone in the space. There is a buzzer-entry system to gain access to the suite which helps us monitor the flow of people into the centre. There is a receptionist who greets anyone who enters the centre. Although BTC follows a harm reduction model, women are not permitted to be actively using substances on-site or to be impaired as a result of substance use. Male partners are not allowed to wait inside the centre for women while they access programming. These safety guidelines around safety were developed based on feedback received directly from women during the early stages of the program. Women were pleased to be asked their opinions about how the program could help them to feel safe. Over the years, women continue to comment and agree that these features are integral to their feelings of safety, trust, and confidence in working towards their intervention goals.

Finally, BTC programming is directed towards women who are mothers; therefore, the physical environment is designed for the children, aged 0 to 6 years, who are in the centre and accessing services. Although it is beyond the scope of this paper to describe how parenting and early intervention services are integrated into treatment for each family, supporting women in their maternal role, while also recognizing and treating them as women, is an important contributor to the success of the program. As women experience modeling and scaffolding to repair, reintegrate, and promote their neurological development, sense of self, and relationship capacity, they become aware of and are able to provide teachings around these factors with their young children. In this way, the intergenerational cycles of IPV and substance use, which have pervaded their own lives, are broken.

### 2.3. Encouraging Regulated Interactions

*The flexibility of this group is that we all get to learn things but also, there are times when women come in here and they’re up to here, ready to cry, and if it’s another group, there’s no way everyone would stop for them, they wouldn’t just stop for that one person, they would keep going. But here you can stop, just for that one person...each person is understanding. Like that’s the whole thing about being an addict and understanding that, not just as pregnant women but as addicts you need to come in here and express yourself when you need to.*

[6] (p. 54)

Self-regulation is promoted as women interact with other clients and with staff at the centre. During the orientation process, women meet with a counsellor who describes the environment of safety and respect at BTC, including the expectations about how everyone in the program is to be treated. For instance, women are asked not to refer to themselves, or others, by “street” names; bullying and gossiping are not tolerated. There are visual reminders in the form of inconspicuous but well-placed signs which remind women to respect the confidentiality of others.

At BTC, staff are conscious of and careful to model regulated interactions with women, other staff members, and external community service providers. Women have frequently commented on how they experience warm and caring relationships with their counsellors, which are different than those they have experienced before, even with family and friends. At the same time, women are given a clear understanding that these are professional and not personal relationships. Women at BTC typically have many other service relationships to maintain, including those with mandated sectors. Having the ability to draw upon the style of regulated behaviour that they observe, as well as the support of their counsellors, is an important part of managing difficult relationships, specifically when those interactions trigger traumatic feelings from their past. Eventually, with support and practice, women are able to navigate personal and professional relationships outside of BTC in a healthy manner that promotes the achievement of their goals. The following quote was obtained from an interview with a BTC client for the purposes of program evaluation in 2014. Informed consent was provided by the client:

I try to get everybody on the same page. Like here, I learned that from here. Everybody knows. The workers here have their meetings on Friday and it’s a team…everybody is a team. So I got used to that. Everybody’s on the same page. I like the daycare to be on the same page as the school. I like the resource centre to be on the same page as everybody…children’s aid…everybody has to be on the same page for the kids.

At BTC, case conferences are often held with a woman and representatives from all of the agencies that provide services to her family. These services may include her parole officer and/or her child protection worker. Typically, the first time a case conference is held, the woman is understandably nervous and hesitant to share her opinions or to speak during the meeting. Through the support and example of her BTC counsellor, the woman is able to see the benefit of describing, not only her challenges, but also her own personal goals and successes with all service providers. Over the course of a few meetings, she may be able to see the benefit of a shared view of her family situation and eventually become comfortable enough to chair the meeting herself.

### 2.4. Attention to Readiness and Internal Processes

*They didn’t force anything upon you like “Okay you have to go to twelve step meetings, or you have to go to (detox), or you have to look at it this way”. They give you, here’s your five options, check every one out if you want, or you can choose which one, which path you want to go, but everyone’s an individual and everyone’s different, and one way may work for me that doesn’t work for (another client)…Everybody’s an individual, it’s not like “Here’s how you’ve got to do it, and everyone’s got to do it this way, and if you don’t do it this way you’re going to fail…” or they didn’t ever put it that way. It was always, “Here’s your options, and try which ways you would like to do it in”, not someone telling you “You have to do this”.*

[6] (p. 84)

At BTC, the Transtheoretical Model of the Stages of Change [17] is used to support women to consider change, not only in regard to their substance use, but also in other areas where they have developed goals, including interpersonal relationships, education or vocational aspirations, parenting and/or their children’s development and well-being. The model recognizes that women require different types and levels of support based on their readiness to work towards their goals of making change. Programs and services at BTC are mapped onto the stages of change (precontemplation, contemplation, preparation, action, maintenance, and relapse) that women are demonstrating in different areas. For example, when women enter the program, they are typically very early in their consideration of making change in their substance use. Even if they are no longer regularly using their identified primary substance, there may be other substances that they are using in a problematic manner. The *Relapse Prevention Group* is recommended for women who are in the precontemplation or contemplation stage of change with their substance use. Women who are closer to the action stage often participate in the *Connections* group, which is a program developed at BTC to address IPV and its connection to substance use and parenting [18], and women in the maintenance stage can participate in the *Recovery* group. Understanding and assessing women’s readiness for change is critical in engaging women in the process of change.

Counsellors at BTC use diverse strategies to support women in developing their capacity to regulate and meet their intervention goals. Self-regulation is supported through attention to and identification of feelings and internal states. Counsellors support women to identify, name, and reflect on internal emotional states, which may be their first experience in being helped to understand and cope with feelings. Noticing and reinforcing when a woman is able to regulate in challenging situations is as important as providing strategies when she is having difficulty. Grounding and mindfulness strategies are also used to support regulation and recovery. Milligan and colleagues [10] have outlined a similar range of treatment strategies for substance using women, including helping women to develop action-oriented recovery goals, supporting the navigation of community resources and systems, as well as understanding and orienting supports to a woman’s own learning style. 

## 3. Promoting Safety and Capacity in Relationships

Not only can early exposure to IPV negatively impact the development of self-regulation, but IPV also affects the development of relational capacity. From a developmental-relational perspective, early experiences of violence in relationships can disrupt the positive bonds between children and their caregivers, and damage children’s sense of safety in relationships [19,20]. These relational disruptions can have long-term consequences, including developmental cascades that impact adults’ difficulties forming healthy relationships, difficulties in parenting, ineffective coping strategies, and problematic substance use. As such, forming trust with women who have a history of IPV in order to promote safety and enhance ongoing relationship capacity requires systemic and relational considerations in service provision, as well as attention to an open and welcoming therapeutic environment. In this section, we will discuss two specific ways in which safety and relationship capacity are supported at BTC: (1) engagement and building trust in relationships, and (2) maintaining relationships.

### 3.1. Engagement: Building Trust in Relationships

*There is obvious communication between all the workers here… a real open, honest, communication, so you know I can talk to anyone of the women here and feel that same sort of support and you know that if it’s necessary they’ll probably pass on that information to someone else so everyone’s on the same page as to where I’m at.*

[6] (p. 86)

At BTC, the formation of that trust begins in the very first appointments with the intake counsellor. Women are welcomed into the centre and given a tour that includes access to food and transportation costs, components which have become important for early engagement. Ensuring informed consent is a foundational part of the relationship development. There is transparency around service partnerships and practices, the voluntary nature of the program, the role of research at BTC, expectations that women should have around privacy and confidentiality, as well as the limitations of our services. At the beginning of the therapeutic relationship, having open and honest discussions with women creates a foundation of trust and provides an opportunity for repair in relationships when challenges arise.

Other systemic considerations that promote safe relationships include a requirement that staff at BTC are trained in trauma-informed practice [21]. Efforts are made to reduce the barriers that women experience in accessing services to meet their own needs, such as providing child-minding and instrumental supports. Finally, women experience a comprehensive and community-based approach to services, with providers from different programs and sectors forming effective and collaborative relationships to support women in reaching their goals.

### 3.2. Maintaining Relationships

*I’ve been with her (BTC counsellor) when I was clean, when I relapsed, when I went through treatment … she’s loving, she’s caring, she’s compassionate, she’s understanding, she’s patient, she’s challenging when need be. She has no bias, she has no judgement, she has resources.*

[6] (p. 53)

*Like she really does listen to you. She listens to what you say. And she remembers. And I assume that she probably talks to all of us, but when she comes to talk to you, she remembers what you said last week and it’s not written down in a book. She knows you as a person.*

[6] (p. 53)

When women who use substances experience programming and therapeutic relationships that are congruent with their needs, it can be a transformative experience that promotes enhanced capacity for relating. Women have described elements of their relationships with BTC counsellors that they felt were facilitative for them. These elements included respect, understanding, authenticity, mutual empathy, and reciprocity [8].

Staff at BTC recognize that attending to women’s emotional safety sometimes requires crisis management—meeting the woman where she is at and managing her immediate needs and concerns before turning the focus to intake forms and assessments. Pacing the collection of current and historical information about a family is critical as well. Some women who use substances can be mistrustful of service providers and are not willing to disclose meaningful information until they have established a sense of trust with a counsellor. Other women seem less able to keep themselves safe and may disclose information too quickly, which can leave them feeling exposed and vulnerable after the appointment. This may trigger service avoidance or substance use as a coping mechanism. Supporting a woman to provide information at a slow pace can help her to stay emotionally safe and understood. Similarly, BTC staff members do not view the intake assessment as a process to uncover the “truth” about a woman’s life. A woman’s story unfolds over time and she should not be required or encouraged to disclose more than she is ready to, especially related to her history of trauma. For this reason, counsellors at BTC use a narrative approach when they are establishing relationships with women and learning about their story, as opposed to sitting with an intake questionnaire or going through a chart in a prescribed manner. 

Motivational interviewing approaches are designed to support women to build commitment towards the changes they need to make to reach their stated goals; it also promotes understanding of their own ambivalence to these changes [22]. Through a therapeutic relationship with a counsellor who is empathic, accepting, and non-judgmental, external barriers to change are identified and the benefits to making change are explored and highlighted. Motivation and ultimately success in making change are determined by the interaction between the woman and her counsellor; however, the locus of control for change belongs to the woman and not to program staff. This approach is a key feature of the work that occurs at BTC. Through this relational intervention strategy with women, staff recognize and reflect that the experience of interactions within BTC may feel significantly different than those that women have had previously in both service and personal relationships. With that in mind, BTC staff are patient and supportive as women attempt to make changes in their interaction styles and in their ability to trust in relationships. BTC becomes a place where women practice a new style of behaving and relating, often for the first time. BTC staff are aware that women are constantly observing interactions that staff have with other clients, between each other, and with external community service providers as a model of healthy relationships; women look to staff to support the scaffolding of positive interaction styles. Finally, through their experiences of healthy interactions with staff, women who attend programming at BTC eventually begin to trust and depend on the knowledge that their stories will be held and remembered, even between counselling sessions. Women’s awareness that they are kept in the mind of their therapists supports the repair and reintegration of a healthy sense of self and promotes a positive capacity for relationships for women who have been impacted by IPV and who use substances.

## 4. Conclusions

In this paper, we outlined specific strategies used at BTC, which promote self-regulation, executive functions, safety, and relationship capacity, in the context of a community-based prevention and early intervention program. A large part of the success of BTC has been based on the fact that it is a small, community-based, relational program. That being said, one challenge has been balancing the importance of the scale of the service model with the scope of issues faced by women with substance use issues, trauma, and histories of interpersonal violence. Similarly, women at BTC have indicated that there is a need for more services like BTC in other neighborhoods and communities, and that BTC should have more outreach and promotion in the community. In the future, it will be important to consider replications of the model described in this paper across jurisdictions, that are based in each individual community and that would maintain the fidelity of the developmental-relational aspects of the program.

Using a developmental–relational approach helps us understand the link between experiences of violence in relationships across development and later substance use issues. These factors can perpetuate the pathway of IPV across development, while reinforcing substance use as a necessary means of coping. At BTC, we are learning that it is possible to engage in a process of reparation and reintegration for those who have experienced IPV across development, by using a developmental-relational approach to guide care for women. Supporting a multi-generational response to issues of women’s substance use recognizes the fact that women who misuse substances were once (often) children who experienced trauma within relationships, and that many women are also mothers striving to break this cycle in order to make their children’s lives different. As women at BTC have shared, breaking the cycle of substance use involves listening to children who are abused or neglected, as that is where their problematic trajectories of substance use began. This quote was obtained from an interview with a BTC client as part of a research study in 2016. Informed consent was provided by the client:

As amazing as it is to have these programs when we’re 25, 26…if we can get the girls even younger. In my experience talking to women who have been raped [as children], there was nobody there to listen to them. Nobody believed them…I think if we can try to have a voice for the younger generation we can somewhat break the cycle of it not turning into a 10 year issue of drug use or abuse.
